# A decade of *GigaScience*: the importance of community organizations for open and FAIR efforts in neuroinformatics

**DOI:** 10.1093/gigascience/giac060

**Published:** 2022-06-14

**Authors:** Maryann E Martone

**Affiliations:** University of California, San Diego, Neurosciences, 9500 Gilman Drive, La Jolla CA 92093, USA; San Francisco VA Medical Center, 4150 Clement St, San Francisco, CA 94121, USA

## Abstract

Neuroscience has undergone a significant transformation over the past decade, becoming an increasingly open and FAIR discipline. I provide personal perspectives on the importance of two community organizations, FORCE11: The Future of Research Communications and e-Scholarship and INCF: The International Neuroinformatics Coordinating Facility in providing the intellectual and community environment where ideas and open sharing of data and code were incubated and tried.

## Background

Congratulations to *GigaScience* for 10 years of being on the forefront of open science and innovative publishing! I appreciate the opportunity to share some of my thoughts about the profound transformation in science in general and neuroscience in particular over this same time period towards open and FAIR. For those of us working in the trenches of open science, it is easy to think that the rate of change is too slow.  At a recent workshop at the US National Academies of Science, Engineering and Medicine on changing the culture of data management and sharing [1], some claimed little had changed over the past decade. True, we were talking about the same topics-data sharing, data citation, incentives-yet the overall feeling was one of optimism. Why? We were gathered ahead of the launch of the US National Institutes of Health (NIH) sweeping new data policy in 2023 which mandates wholesale data sharing across biomedicine.  Ten years ago, we'd have been talking about how to convince scientists that data sharing is good;  now it is required.  Ten years ago, a lot of experiments like *Gigascience* were being launched; a lot of infrastructure was being built.  Some succeeded and some failed.  But the groundwork for how we could and should go about sharing data was laid, in fits and starts and through hard lessons learned.  We are not starting from nothing. Ten years ago, it was the wild west when it came to how data resources were built. Now  “FAIR: Findable, Accessible, Interoperable and Reusable is providing a coherent vision for data resources.

### So how did we get to this point?

I want to highlight two community organizations that were instrumental in developing the ideas and infrastructure propelling this transformation:  FORCE11.org, the Future of Research Communications and eScholarship, and the International Neuroinformatics Coordinating Facility (INCF.org). Both of these unique organizations provided the intellectual and community environment where a future of data and code sharing were envisioned and realized. Indeed, the first time I heard about *GigaScience* was at the second FORCE11 *Beyond the PDF* conference in Amsterdam 2013.

I often get the feeling that every day since the internet became intertwined with our existence some scientist wakes up and asks:  “Why are we still publishing static pdf files when we could do so much more?”  In 2011 a group of people asked that question at the first *Beyond the PDF* conference held at the University of California, San Diego. It remains the most electrifying conference I ever attended.  People were passionate about the possibilities of open science and the new technologies available for transforming scholarly communication. People were also angry at the abuses of our current publishing and reward system that allowed so much work to be published behind a paywall in inflexible formats.  Many like myself had not questioned the “bizarre triple pay” system of scientific publishing before then [2]. After that, it was impossible not to. FORCE11 was founded one year later to harness this incredible energy towards transforming scholarly communications through technology.

At that conference, I spoke about our work in the Neuroscience Information Framework (NIF), a project launched in 2008  to survey and catalog all of the new types of products being produced in neuroscience-code, software platforms, datasets and databases [3]. NIF's job was to catalog them, find a way to query across them and make recommendations on how to make them better. There were already hundreds of such resources available for neuroscience, thanks largely to early investments by the US National Institutes of Health Human Brain Project (USHBP) starting in the mid-nineties 4, but they were difficult to find, access and use. In our presentations, we coined “NIF's rules for data.”  You have to be able to: 1)  Find it;  2)  Access it;  3)  Understand it.

### Making neuroinformatics more FAIR

You can hear in these rules faint echoes of the FAIR data principles, published about 5 years later. FAIR neatly conceptualized what we and others faced when attempting to work across and within databases. The declaration that data should be FAIR: Findable, Accessible, Interoperable and Reusable along with the 15 recommendations for doing so arose from a workshop in Leiden, was posted on FORCE11 in 2014 [5] and published in 2016 [6].

FAIR seemed to gain wide adoption almost immediately. The acronym was brilliant, lending itself to innumerable plays on words and explicitly expressing a value judgment: who does not want to be FAIR?  But more importantly, FAIR incorporated many of the lessons learned over the early years in trying to gain adoption for new approaches. FAIR is: 1)  Simple-not a 50 page specification but ∼20 lines of text;  2) Flexible, laying out goals not a specific technological approach; 3)  Respectful of community norms, delegating the specific details to individual scientific communities to interpret as required; 4)  Aspirational, all did not have to be implemented at once to improve data.

Their issuance also coincided with a burgeoning recognition by funders, scientists and journals that open data was required to fuel new opportunities in data science and to combat growing concerns about reproducibility and transparency. Interestingly, the FAIR principles themselves are agnostic with respect to open science, yet they are an integral to it. If data can't be found, accessed, and reused then what does it matter whether or not it is open? The FAIR principles are largely directed towards those who are providing the data repositories and associated tools for hosting and sharing open data and code. Ask a bench scientist about FAIR criteria such as persistent identifiers and you will likely get a blank stare. They have no opinion as to whether these are good things or not. On the other hand, ask them about data sharing and open science, and you may get an earful. Neuroinformatics, at least the branch concerned with building information architectures for digital neuroscience, can trace its roots back to the mid 1990s and the US HBP. At that time, overall attitudes towards open neuroscience were decidedly negative. A dedicated core of open neuroscience databases and proponents came out of the US HBP, but for the most part, mainstream neuroscience responded with disinterest, skepticism, resistance and outright hostility [7]. I was asked to speak about data sharing to a meeting of journal editors circa 2013, and the dominant attitude was expressed as an expletive!

But fast forward only a few years later to the launch of the large international brain initiatives in the EU, Japan, the US, Canada and China all of which recognized that open and effective data sharing was the only way that neuroscience could mobilize the resources and manpower to solve the mysteries of the nervous system [8]. The rest of neuroscience is following, as many journals and major funders are now requiring data sharing. And it is because of community organizations like FORCE11 and the INCF that when neuroscience was ready to move towards open and FAIR, the necessary human and technical expertise was there to support it.

### Exemplars of community organizations: FORCE11 and INCF

FORCE11 provided much of the early impetus towards envisioning alternate forms of scholarly communication through FAIR and other efforts like the Joint Declaration of Data citation Principles [9]. However, these ultimately need to be interpreted and implemented within a specific discipline to have impact and that is where community organizations are critical. INCF was uniquely situated to play the role of community coordinator for FAIR neuroscience. The INCF was launched in 2005 as an international organization dedicated to promoting the sharing of neuroscience data through the coordination of infrastructures and standards. INCF provided a home to develop the nascent field of neuroinformatics after the US HBP ended. Through its early efforts to bring neuroscientists together to work communally on technology and standards, it grew a community of practitioners who learned to work in an open, collaborative manner across international boundaries to define standards and approaches to sharing and integrating neuroscience data. INCF is proud that its members developed and/or run many of the infrastructures for the large international brain initiatives as well as many of the foundational infrastructures serving worldwide neuroscience.

Why are these organizations so important?  They provide a sustained forum for the important discussions to occur, approaches to be tried and lessons to be learned and disseminated. The conferences cover a range of topics, from the technical to the sociological, ensuring that the technology is matched to larger applications. Their members are characterized by open and community oriented views, where information is exchanged freely and both personal and professional advancement are tied to greater scientific and societal good. They bring together multiple stakeholders who normally do not network together-librarians, publishers, tool builders, researchers, commercial providers- but who often bring valuable knowledge to the larger problem. They support the community-initiated working groups where participants learn to work collaboratively and which serve as incubators for future leaders.  Without these working groups, a lot of the ideas hatched at the conferences would die on the vine.  I am grateful to both of these organizations for my own professional development and the lively, intellectually stimulating, open and collegial atmosphere they provided. I now find it difficult to work in any other way.

## Conclusions

So have all the challenges of open and FAIR science been solved?  Of course not.  The current form of scholarly communication through journals has been refined for over 350 years;  we've been at this for 25. Issues in data citation, metrics, skills and sustainability have yet to be solved completely.  Increased attention must be paid to the usability not only of the data but the infrastructures themselves, a priority of the INCF.  But the only way to develop a functioning system is to get going. Although some may look at the new mandates as a burden, we are all being asked to participate in defining an entirely new way of communicating science and that is exciting. I hope you'll consider joining your fellow pioneers in FORCE11 and INCF to help bring us there.

## Abbreviations

FAIR: Findable, Accessible, Interoperable and Reusable; HBP: Human Brain Project; FAIR: Findable, Accessible, Interoperable and Reusable; FORCE11: Future of Research Communication and e-Scholarship; INCF: International Neuroinformatics Coordinating Facility; NIF: Neuroscience Information Framework.

## Data Availability

Not applicable

## Declarations

### Competing interests

Dr. Martone is a founder and has equity interest in SciCrunch Inc., a start up company out of UCSD that provided tools in support of Research Resource Identifiers, the rigor and reproducibility.

## Funding

Not applicable

## About the Author

Maryann Martone received her BA from Wellesley College in Biological Psychology and Ancient Greek and her Ph. D. in Neuroscience from the University of California, San Diego. She is a professor Emerita at UCSD, but still maintains an active laboratory, the FAIR Data Informatics Lab. She started her career as a neuroanatomist, specializing in light and electron microscopy, but her main research for the past 20 years focused on informatics for neuroscience, i.e., neuroinformatics. She led the Neuroscience Information Framework (NIF), a national project to establish a uniform resource description framework for neuroscience, and the NIDDK Information Network (dknet), a portal for connecting researchers in digestive, kidney and metabolic disease to data, tools, and materials. Dr. Martone is past President of FORCE11, an organization dedicated to advancing scholarly communication and is the current chair of the Governing Board of the International Neuroinformatics Coordinating Facility.

## Editor's note

This commentary is part of a series to celebrate a Decade of GigaScience, to coincide with the 10th anniversary of our launch in July 2012. These papers take a look back at 10 years of advances in large-scale research as open science has become mainstream.
